# Effect of modified CEP-3 diluents with aqueous soybean extract on liquid semen quality in Ongole crossbred bull

**DOI:** 10.14202/vetworld.2023.1075-1083

**Published:** 2023-05-17

**Authors:** Dian Ratnawati, Kuswati Kuswati, Aulia Puspita Anugra Yekti, Gatot Ciptadi, Sri Rahayu, Trinil Susilawati

**Affiliations:** 1 Research Center for Animal Husbandry, Research Organization for Agriculture and Food, National Research and Innovation Agency of The Republic of Indonesia (BRIN), Cibinong Sciences Center, Bogor, Indonesia; 2 Department of Animal Reproduction and Breeding, Faculty of Animal Science, Universitas Brawijaya, Indonesia; 3 Department of Livestock Production, Faculty of Animal Science, Universitas Brawijaya, Malang, Indonesia; 4 Department of Biology, Faculty of Mathematic and Natural Science, Universitas Brawijaya, Malang, Indonesia

**Keywords:** aqueous soybean extract, liquid semen, Ongole crossbred cattle

## Abstract

**Background and Aim:**

Egg yolk (EY) is commonly used as an extracellular cryoprotectant in semen diluents but has some negative effects. Therefore, this study aimed to assess the potential of lecithin derived from plants, such as soybeans, as an alternative extracellular cryoprotectant and to characterize liquid semen quality of Ongole crossbred bulls using a modified caudal epididymis plasma-3 [CEP-3 (m)] as a base diluent and aqueous soybean extract (ASE).

**Materials and Methods:**

A bull with progressive motility (PM) of fresh semen >70% was used. Two soybean extracts were also used, namely, ASE 1 and ASE 2, obtained by extraction procedures 1 and 2, respectively. The study was conducted using an experimental design with 11 treatments and ten replications, with diluents comprising different levels of ASE 1 and ASE 2, as well as a positive control with 10% EY. The parameters measured were motility (M) and its kinetic parameters, including PM, M, velocity curve linear, velocity straight linear, velocity average pathway, linearity, straightness, wobble, amplitude lateral head beat cross frequency, and hyperactivity using computer-assisted sperm analysis, viability, and spermatozoa abnormalities.

**Results:**

The CEP-3(m) diluent formula and ASE 1 at a 30% level maintained the PM of spermatozoa up to day 5 (40.7% ± 16.1%) of cold storage. Meanwhile, the CEP-3(m) diluent formula and ASE 2 could only maintain PM >40% until day 3 (42.1% ± 13.5%) of cold storage at a 30% level. The CEP-3(m) diluent and ASE 1 at a level of 25%–30% supported spermatozoa life (viability) up to day 5 with a value >80% (81.8 ± 3.5; 86.4 ± 2.6). The abnormality value of spermatozoa in various diluents during cold storage on days 0–5 was below 20%.

**Conclusion:**

Soybean extracts 1 and 2 can substitute EYs as extracellular cryoprotectants in modified CEP-3 basic diluents. Soybean extract 1 can support the life of spermatozoa up to day 5 but may cause the viscosity and movement of spermatozoa to be hyperactive. Soybean extract 2 can support the life of spermatozoa up to the 3^rd^ day of cold storage and produces progressive (non-rotating) movement patterns. Further, research is recommended with higher levels of ASE 2.

## Introduction

The application of artificial insemination using liquid semen offers many advantages, including not requiring expensive infrastructure during implementation, not requiring liquid nitrogen for storage, supporting good quality spermatozoa (up to 10 days) [1], being easy and simple to process and being safer [2]. Semen diluents play an essential role in the fulfillment of physiological requirements, such as being iso-osmotic, having a metabolizable substrate composition, with a pH close to neutral, containing intracellular and extracellular cryoprotectants, buffers, energy sources, and antibiotics (antibacterial) [3–5]. An optimal diluent composition is expected to protect spermatozoa from damage caused by cooling, such as cryodamage or cryoinjury [6–8]. Liquid semen is considered successful if the decrease in motility (M) and metabolic activity due to cooling can be reversible [9].

The function of a cryoprotectant is to protect spermatozoa from cold shock and ensure the maintenance of membrane integrity. Egg yolk (EY) is an extracellular cryoprotectant that contains lecithin and is frequently used in cryopreservation. However, concerns have arisen about its use in semen diluent due to its potential to spread zoonotic diseases and adversely affect the quality and fertility of spermatozoa. There are several negative effects of EY in diluents, including bacterial contamination (exotic diseases) [10, 11], reduction in spermatozoa fertility by bacterial endotoxin, and interference with a microscopic evaluation due to the presence of debris/vacuole, consisting of varied composition that is difficult to control, can alter the structure and integrity of spermatozoa membranes, and be antigenic in the blood circulation and reproductive tract [12–15]. An assessment is required to determine the suitability of using lecithin derived from plants, including soybeans. Soybean is a source of lecithin free from animal elements and can function as an extracellular cryoprotectant, substituting EY [7, 16–18]. Commercial soybean lecithin, such as Biochipos plus^®^ (Tiefenbach, Germany), Bioexcell^®^ (L’Aigle, France), and Andromed^®^ (Tiefenbach, Germany), is readily available. Soybean lecithin can be used as a diluent for liquid and frozen semen in various animals, such as bulls, goats, buffaloes, and rams. Studies have shown that using soy lecithin in the frozen semen of buffalo can effectively support the quality of spermatozoa on the 5^th^ day of storage [19]. Furthermore, its use in frozen semen at 1%–1.5% and 10% for buffalo produced the best results in supporting the quality of spermatozoa [20, 21]. Soy lecithin-based diluents have been shown to produce the same quality as EYs [22]. The phosphatidylcholine and several fatty acids, including linolenic, linoleic, oleic, stearic, and palmitic acids in soybean lecithin, play a role in preventing damage to the plasma membrane of spermatozoa due to the dilution, cooling, and disposal processes [14, 23–25]. The mechanism of spermatozoa protection is by forming a coating film on the membrane, thereby preventing damage [22, 23]. It also supports the structural stability of spermatozoa cells, which have a specific fat composition with a high ratio of polyunsaturated fatty acid, and protects mammalian phospholipid membranes [26, 27]. Liquid ram semen with a diluent of tris, fructose, and citric acid combined with commercial soybean extract could maintain progressive M (PM) for up to the 8^th^ day of storage [5]. Meanwhile, the study by Immelda *et al*. [28] found that the use of 15% soybean juice diluent could only support the viability of the spermatozoa of Sapudi sheep for 24 h of storage, with a percentage >80%. Similar results were also observed in a study on buffalo semen conducted by Singh *et al*. [4], where the 25% level of soy milk-based spermatozoa diluent could only support the M of individual spermatozoa up to the 48^th^ h with a value >40% and viability exceeding 80%, which could only be achieved at the 24^th^ h of storage. A previous study on bovine semen by Sugiarto *et al*. [29] stated that the combination of CEP-2 diluent and 10% soybean extract could maintain spermatozoa M (>40%) in Limousine cattle up to 24 h of cooling. However, the viability of spermatozoa at 0 h of cold storage could not reach 80%. The combination of tris diluent and soy extract 3% and 5% could support sperm M of Limousine cattle up to the 4th day of cold storage, but viability >80% could only be achieved on the 1^st^ day of storage [30]. This is in contrast to a study that stated that the use of tris diluent and 5% soybean extract could support spermatozoa M (>40%), viability >80%, and spermatozoa abnormalities <20% only shortly after mixing with fresh semen of Kebumen cattle [31].

Studies on the use of whole soybean extract as a 100% substitute for EYs have never been conducted. The potential use of whole soybean extract is expected to provide an alternative to the use of plant-based extracellular cryoprotectants. Therefore, this study aimed to characterize the liquid semen quality of Ongole crossbred bull in a modified CEP-3 as a based diluent and extracellular cryoprotectant using aqueous soybean extract (ASE).

## Materials and Methods

### Ethical approval

All experiments were approved by the Animal Ethics Committee of the Indonesian Agency for Agricultural Research and Development (Balitbangtan/Lolitsapi/Rm/01/2022).

### Study period and location

The study was conducted during January and-February 2022. The study was conducted in Pasuruan, East Java, Indonesia.

### Place and time of research

This study was conducted at the Beef Cattle Research Institute (Pens and Reproduction Laboratory). An Ongole crossbred bull, aged 5–6 years, with a body weight of 400–500 kg, and fresh semen quality with PM over 70% was used. The bull used was one from which semen had been routinely collected twice a week using an Artificial Vagina, and libido optimized with false mounting.

The design of this study was a completely randomized design with 11 treatments and ten repetitions. Treatments administered include T1 = CEP-3(m) + ASE 1 10%, T2 = CEP-3(m) + ASE 1 15%, T3 = CEP-3(m) + ASE 1 20%, T4 = CEP-3(m) + ASE 1 25%, T5 = CEP-3(m) + ASE 1 30%; T6= CEP-3(m) + ASE 2 10%, T7 = CEP-3(m) + ASE 2 15%, T8= CEP-3(m) + ASE 2 20%, T9 = CEP-3(m) + ASE 2 25%, T10 = CEP-3(m) + ASE 2 30%, and T11 = CEP-3(m) + 10% EY.

### Extraction procedure 1

A total of 25 mg of soybeans were mixed with 1% NaHCO_3_, soaked in hot water, and then removed from the husks. The soybeans were ground with 125 mL of freshly boiled water and filtered. The dregs obtained were then ground with 125 mL of hot water, filtered, and centrifuged. The obtained filtrate was centrifuged, and the top layer was placed into a tube and filtered (modified by Immelda *et al*. [28] and Sartini *et al*. [32]).

### Extraction procedure 2

A total of 100 mg of soybean were cleaned and soaked in water. The soybean was drained and placed in warm water for 10 min. It was then drained again, ground with 200 mL of distilled water, and filtered. The obtained filtration was placed into a glass tube and centrifuged at a speed of 1008× *g*. The top layer was then ready to be used (modified by Da Costa [33]).

### Preparation of CEP-3 (modified) diluents

The modified CEP-3 was composed of NaCl 0.09 mg, KCl 0.05 mg, CaCl_2_(H_2_O)_2_ 0.04 mg, MgCl_2_(H_2_O)_6_ 0.08 mg, NaHCO_3_ 0.1 mg, NaH_2_PO_4_ 0.11 mg, KH_2_PO_4_ 0.27 mg, fructose 0.27 mg, Tris 1.62 mg, citric acid 0.82 mg, penicillin 0.006 mg, streptomycin 0.01 mg, and sterile distilled water 100 mL [32]. All ingredients were mixed and homogenized with a stirrer for 30 min. The pH of the diluent was then adjusted (±7) by adding HCl if the pH was too alkaline and NaOH if the pH was too acidic.

### Processing of liquid semen

The fresh semen obtained was diluted with the prepared diluents. A total of 25 μL of fresh semen was added to 1 mL of diluent in a test tube, slowly homogenized, and covered with a plastic wrap. The test tube was then placed in a glass beaker that had been filled with water (water jacket), as modified by Indriani and Wahyuningsih [34]. It was then stored in a refrigerator at a temperature of 3°C–5°C (cold storage). The quality of liquid semen was observed every day until the 5^th^ day.

### Spermatozoa M analysis (CASA)

A total of 3 μL of the semen was taken using a pipette, placed on an object glass, covered with a cover glass, and placed on a stage warmer. Observations were made using a magnification of 20× and 10×, and three fields were taken for each sample [35].

### Examination of viability and abnormality of spermatozoa

A semen smear was made on an object glass by mixing one drop of semen with one drop of eosin-nigrosin. Examination of the semen smear was carried out using a 1000× magnification microscope. A total of 100 spermatozoa were evaluated, with dead spermatozoa appearing red and live spermatozoa appearing transparent [36]. The percentage of spermatozoa viability was calculated by dividing the number of live spermatozoa by the total number of spermatozoa observed (live and dead) and multiplying by 100% [37]. A similar procedure was conducted to calculate the percentage abnormality of spermatozoa. The total of abnormal spermatozoa was divided by the total number of spermatozoa observed (normal and abnormal) and multiplied by 100% [37].

### Parameters and data analysis

The parameters measured were the M of spermatozoa and their kinetic parameters, including M, PM, velocity curve linear (VCL), velocity average pathway (VAP), velocity straight linear (VSL), linearity (LIN), straightness (STR), wobble (WOB), beat cross frequency (BCF), amplitude lateral head (ALH), hyperactivity (H), spermatozoa viability, and abnormalities. Data were analyzed using analysis of variance with the Statistical Package for the Social Sciences 16.

## Results

The results of observations of the M and PM of liquid semen spermatozoa during cold storage are listed in Table-1. It was observed that PM and spermatozoa M decreased during cold storage in all diluent formulas. This decrease was faster in CEP-3(m) + ASE 2 diluent than in CEP-3(m) + ASE 1 and CEP-3(m) + EY 10%. The CEP-3(m) diluent formula and ASE 1 at the 30% level maintained the PM of spermatozoa until the 5^th^ day (40.7 ± 16.1%) of cold storage. Meanwhile, the CEP-3(m) diluent formula and ASE 2 could only maintain PM above 40% until day 3 (42.1 ± 13.5%) of cold storage at the 30% level. The CEP-3(m) + ASE 1 and ASE 2 formulas with levels below 30% resulted in lower PM. The performance of the CEP-3(m) + EY 10% diluent was significantly higher than the other formulas, with PM reaching 57% on day 5. Spermatozoa M in diluent CEP-3(m) + EY 10% was significantly higher than in the other formulas. The diluent formula CEP-3(m) + ASE 1 at levels 30% and 25% resulted in M values of 63.1% and 43.6% on day 5, respectively.

**Table-1:** Progressive motility and motility of spermatozoa of liquid semen with various diluent formulas.

Progressive motility	Days of cold storage					

	0	1	2	3	4	5
T1	72.7 ± 5.4^a^	41.7 ± 21.8^a^	16.2 ± 13.1^a^	8.4 ± 10.7^ab^	1.4 ± 1.4^a^	1.3 ± 1.5^a^
T2	73.7 ± 5.9^a^	53.9 ± 15.8^b^	27.8 ± 17.1^ab^	28.4 ± 17.1^cd^	14.1 ± 11.0^bc^	6.2 ± 5.2^ab^
T3	75.1 ± 4.1^a^	67.1 ± 3.6^cd^	59.8 ± 9.0^ef^	40.3 ± 17.3^f^	21 ± 14.7^cd^	12.2 ± 6.7^b^
T4	78.2 ± 6.0^ab^	73.7 ± 3.7^de^	67.2 ± 8.4^fg^	57.3 ± 7.8^f^	39.6 ± 11.8^e^	26.9 ± 15.0^c^
T5	74.3 ± 4.2^a^	73.3 ± 12.0^de^	69.4 ± 5.4^fg^	62.5 ± 7.7^fg^	56.1 ± 8.6^f^	40.7 ± 16.1^d^
T6	82.1 ± 5.6^bc^	41 ± 21.1^a^	16.2 ± 14.1^a^	3.2 ± 3.1^a^	0.5 ± 0.7^a^	0.2 ± 0.5^a^
T7	86.1 ± 5.8^c^	59.9 ± 17.1^bc^	34.7 ± 17.2^bc^	12 ± 10.1^ab^	3.3 ± 2.8^ab^	0.5 ± 0.8^a^
T8	84.9 ± 5.6^c^	68.7 ± 8.8^cd^	45.3 ± 10.0^cd^	19.9 ± 9.2^bc^	5.1 ± 4.2^ab^	2.9 ± 1.5^ab^
T9	83.7 ± 7.3^bc^	73.7 ± 7.8^de^	49.9 ± 17.7^de^	37.2 ± 17.6^de^	18.6 ± 15.2^cd^	6.2 ± 5.1^ab^
T10	86.5 ± 8.3^c^	72.6 ± 8.6^de^	61.6 ± 8.5^ef^	42.1 ± 13.5^e^	25.9 ± 16.0^d^	11.5 ± 7.9^b^
T11	83.5 ± 3.2^bc^	84.1 ± 5.8^e^	79 ± 6.3^g^	72.5 ± 3.6^f^	70.1 ± 7.1^g^	57 ± 9.3^e^
Motility						
T1	89.5 ± 5.1^a^	60.7 ± 23.3^a^	32.5 ± 20.4^a^	17.3 ± 13.8^ab^	5.9 ± 4.2^a^	5.3 ± 3.7^a^
T2	92.8 ± 5.0^ab^	72.3 ± 16.2^ab^	48.4 ± 22.5^bc^	46.8 ± 18.1^de^	28 ± 19.8^bc^	16.7 ± 11.4^ab^
T3	93.4 ± 4.0^bc^	88.7 ± 6.2^cd^	81.6 ± 8.9^def^	61.9 ± 16.6^g^	37 ± 18.1^c^	21.5 ± 12.0^b^
T4	94.8 ± 2.5^bc^	93.9 ± 3.4^d^	87.4 ± 6.2^ef^	81.6 ± 6.3^g^	63.3 ± 13.0^d^	43.6 ± 19.3^c^
T5	93.5 ± 2.3^bc^	90.4 ± 9.2^cd^	90.7 ± 5.9^ef^	84.5 ± 5.2^g^	81.1 ± 8.5^e^	63.1 ± 19.8^d^
T6	95.2 ± 3.6^bc^	60.3 ± 24.8^a^	35.5 ± 24.1^ab^	11 ± 6.7^a^	4.4 ± 2.5^a^	4.2 ± 6.1^a^
T7	95.1 ± 4.1^bc^	77.6 ± 15.6^bc^	52.5 ± 22.0^c^	27 ± 18.1^bc^	11.3 ± 8.3^a^	4.2 ± 3.7^a^
T8	94.3 ± 3.5^bc^	85.8 ± 9.9^cd^	68 ± 11.7^d^	37.6 ± 13.0^cd^	15.2 ± 7.1^ab^	9.7 ± 4.5^ab^
T9	96.3 ± 2.8^bc^	90 ± 5.3^cd^	76.4 ± 15.3^de^	55.1 ± 24.9^ef^	37.2 ± 27.3^c^	17.6 ± 14.2^ab^
T10	97 ± 2.2^c^	89.1 ± 5.4^cd^	81 ± 14.1^def^	64.3 ± 14.1^f^	43.9 ± 23.2^c^	20 ± 14.4^b^
T11	95.2 ± 3.8^bc^	95.6 ± 3.4^d^	95.6 ± 2.8^f^	92.9 ± 4.0^g^	91.5 ± 8.1^e^	83 ± 10.0^e^

Different letter notations in the same column indicate a significant difference (p < 0.05)

Table-2 shows the velocities (VCL, VSL, and VAP) of spermatozoa in liquid semen during cold storage, and it was observed that they decreased in all diluents. On day 5, the VCL, VSL, and VAP values of spermatozoa in the CEP-3(m) + ASE 1 diluent were higher than the CEP-3(m) + ASE 2 diluents on each observation day. The high VCL on the CEP-3(m) + ASE 1 diluent indicated the speed of spermatozoa movement in the curve trajectory and the strength of spermatozoa movement. Velocity on diluent CEP-3(m) + ASE 1 (20%–30%) on day 5 was not significantly different from diluent CEP-3(m) + EY 10%. Meanwhile, VSL on diluent CEP-3(m) + ASE 2 (30%) on day 5 was not significantly different with diluent CEP-3(m) + ASE 1 (25% and 30%) and CEP-3(m) + EY10%. The VAP value in the CEP-3(m) + ASE 1 diluent (20%–30%) was not significantly different from the positive control formula.

**Table-2:** Velocity of spermatozoa (VCL, VSL, and VAP) liquid semen with various diluent formulas.

VCL	Days of cold storage					

	0	1	2	3	4	5
T1	74.6 ± 6.7^abc^	62.6 ± 15.3^b^	42.5 ± 14.0^ab^	40.4 ± 18.1^bc^	24.1 ± 7.3^ab^	22.4 ± 9.6^b^
T2	76.3 ± 6.5^abcd^	70.6 ± 9.6^bc^	58 ± 10.9^cd^	53.8 ± 12.6^de^	44.5 ± 8.7^d^	33.6 ± 11.4^cd^
T3	81.7 ± 10.0^cd^	76.7 ± 7.9^cd^	68.2 ± 6.8^def^	59.5 ± 12.7^e^	50 ± 16.5^de^	51 ± 9.7^ef^
T4	82.5 ± 9.9^d^	79.9 ± 7.3^d^	75.3 ± 9.4^f^	70.5 ± 9.2^f^	60.2 ± 11.9^ef^	55.3 ± 12.0^f^
T5	76.3 ± 7.7^abcd^	83 ± 8.4^d^	69.7 ± 17.9^ef^	72.6 ± 10.1^f^	66 ± 10.6^f^	58.6 ± 12.4^f^
T6	74.1 ± 1.8^ab^	52.4 ± 6.4^a^	33.6 ± 15.1^a^	24.5 ± 14.7^a^	19.3 ± 11.9^a^	8.6 ± 6.6^a^
T7	76 ± 6.1^abcd^	63.3 ± 8.1^b^	49.4 ± 7.1^bc^	36.7 ± 14.1^b^	25.7 ± 9.7^ab^	19.1 ± 12.1^b^
T8	76.1 ± 5.5^abcd^	65.7 ± 3.8^b^	53.6 ± 5.7^c^	42.9 ± 9.0^bcd^	31.6 ± 10.4^bc^	28.7 ± 8.1^b^
T9	72.5 ± 6.3^a^	67.4 ± 6.6^b^	54.4 ± 9.9^c^	54.4 ± 6.0^de^	40.3 ± 13.1^cd^	33.1 ± 8.8^cd^
T10	72.4 ± 6.5^a^	67.9 ± 6.9^b^	60.2 ± 5.8^cde^	51 ± 7.0^cde^	43.9 ± 11.3^d^	42.3 ± 10.7^de^
T11	80.9 ± 4.2^bcd^	90.9 ± 8.1^e^	77.8 ± 10.4^f^	72.5 ± 9.0^f^	67 ± 7.3^f^	57.6 ± 5.0^f^
VSL						
T1	37.9 ± 7.1^abc^	26.3 ± 6.4^ab^	17.9 ± 8.2^ab^	13.6 ± 7.7^ab^	8.9 ± 4.3^ab^	6.6 ± 3.8^ab^
T2	35.2 ± 6.5^ab^	27.9 ± 3.7^abc^	22.6 ± 5.5^bc^	19.8 ± 4.5^cd^	14.8 ± 3.6^cde^	11.7 ± 5.1^bcd^
T3	34.2 ± 5.0^ab^	28.6 ± 7.3^abc^	25.9 ± 3.3^cd^	20.3 ± 2.6^cd^	14.7 ± 4.2^cde^	14.6 ± 2.7^cd^
T4	34.1 ± 5.2^ab^	27.6 ± 4.9^abc^	24.9 ± 2.6^c^	21.1 ± 2.8^cd^	18 ± 1.5^def^	16.1 ± 2.3^cde^
T5	31.5 ± 4.1^a^	27.1 ± 3.9^abc^	22.5 ± 1.8^bc^	21 ± 2.1^cd^	18.9 ± 1.7^ef^	16.9 ± 2.8^de^
T6	41.6 ± 5.6^bcd^	25.6 ± 5.6^a^	15.1 ± 7.8^a^	9.8 ± 7.2^a^	6 ± 5.4^a^	3.4 ± 3.0^a^
T7	47.6 ± 7.4^d^	32.2 ± 6.3^cd^	24 ± 5.7^c^	17.3 ± 9.6^bc^	10 ± 4.9^abc^	6.6 ± 7.1^ab^
T8	47.6 ± 9.6^d^	31.5 ± 5.1^bcd^	27.4 ± 4.7^cd^	20.6 ± 6.9^cd^	13.2 ± 6.3^bcd^	11.2 ± 5.0^bc^
T9	41.9 ± 9.4^bcd^	34.9 ± 6.4^d^	26.4 ± 5.8^cd^	27.8 ± 3.4^e^	19.6 ± 7.4^ef^	14.1 ± 7.0^cd^
T10	43.3 ± 10.9^cd^	32.5 ± 5.7^cd^	30.9 ± 5.6^d^	25.7 ± 5.5^de^	20.7 ± 6.1^fg^	21.2 ± 7.2^e^
T11	41.9 ± 11.3^bcd^	25.5 ± 2.4^a^	26.2 ± 4.1^cd^	24.5 ± 2.5^de^	25 ± 3.0^g^	21 ± 2.5^e^
VAP						
T1	51.8 ± 5.8^a^	40.1 ± 8.3^ab^	28.8 ± 12.3^b^	23.4 ± 12.3^b^	14 ± 7.1^ab^	11.7 ± 7.0^abc^
T2	52.6 ± 5.1^a^	47 ± 6.0^cd^	38.2 ± 8.5^cde^	35.3 ± 9.1^cde^	28.6 ± 7.2^d^	20.4 ± 8.0^d^
T3	53.9 ± 6.9^ab^	48.6 ± 7.7^cd^	45.2 ± 5.4^ef^	37.5 ± 7.3^de^	29.7 ± 10.5^d^	32.4 ± 8.9^ef^
T4	54.4 ± 7.7^ab^	48 ± 6.5^cd^	46.6 ± 6.0^f^	42.1 ± 3.8^e^	37.1 ± 5.4^e^	33.3 ± 6.8^ef^
T5	49 ± 3.5^a^	48.3 ± 4.6^cd^	43.2 ± 5.4^def^	42.7 ± 4.6^e^	39.1 ± 4.7^e^	34.5 ± 6.3^ef^
T6	55.8 ± 4.6^ab^	35.5 ± 6.4^a^	21.5 ± 10.5^a^	14.7 ± 10.1^a^	10.5 ± 6.1^a^	4.9 ± 3.8^a^
T7	60.5 ± 7.0^b^	42.5 ± 6.9^bc^	34 ± 6.0^bc^	23.4 ± 11.4^b^	14.1 ± 6.1^ab^	11.2 ± 10.1^ab^
T8	60.4 ± 9.3^b^	42.2 ± 5.1^bc^	36.2 ± 4.9^cd^	27.8 ± 8.7^bc^	18.5 ± 8.1^bc^	15.7 ± 6.3^bcd^
T9	55.1 ± 8.2^ab^	45.2 ± 7.0^bcd^	35.2 ± 7.1^bc^	36.6 ± 4.6^de^	25.3 ± 9.3^cd^	19.2 ± 6.7^cd^
T10	56.1 ± 10.4^ab^	43.9 ± 4.5^bc^	40 ± 5.6^cdef^	33 ± 5.8^cd^	27.7 ± 7.9^d^	27.7 ± 8.4^e^
T11	61.1 ± 8.6^b^	50.7 ± 4.0^d^	47 ± 3.3^f^	42.9 ± 4.2^e^	41.9 ± 5.4^e^	38 ± 4.0^f^

Different letter notations in the same column indicate a significant difference (p < 0.05). VCL=Velocity curve linear, VSL=Velocity straight linear, VAP=Velocity average pathway

The LIN, STR, and WOB of the spermatozoa in liquid semen during cold storage are listed in Table-3, and the values in all diluents decreased during cold storage. The LIN and STR values of spermatozoa in CEP-3(m) + ASE 2 diluents were higher than in CEP-3(m)+ ASE 1 diluents during cold storage on day 5. This showed that the swimming pattern of spermatozoa in the CEP-3(m) + ASE 2 diluent was more regular and progressive. Meanwhile, the WOB value on day 5 showed that the spermatozoa in the CEP-3(m) + ASE 1 and 2 diluents at levels above 15% were not different from the positive control.

**Table-3:** Linearity, STR, and WOB of spermatozoa liquid semen with various diluent formulas.

LIN	Days of cold storage					

	0	1	2	3	4	5
T1	51.8 ± 10.3^bc^	43.1 ± 9.9^cd^	40.4 ± 7.5^cd^	33.8 ± 14.4^ab^	36.4 ± 11.2^bc^	29.5 ± 10.0^a^
T2	46.3 ± 9.4^ab^	40.2 ± 7.5^bc^	39.1 ± 6.6^bcd^	37.1 ± 2.9^abc^	32.9 ± 3.1^abc^	33.6 ± 6.2^a^
T3	42.3 ± 6.6^a^	37.3 ± 7.6^bc^	38.3 ± 6.0^bcd^	35.1 ± 6.1^ab^	29.9 ± 3.7^ab^	29.1 ± 5.5^a^
T4	41.5 ± 5.8^a^	34.6 ± 6.1^ab^	33.6 ± 4.9^ab^	30.5 ± 6.4^a^	30.9 ± 5.6^ab^	29.9 ± 5.7^a^
T5	41.7 ± 7.4^a^	33.1 ± 6.6^ab^	30.4 ± 3.6^a^	29.4 ± 4.9^a^	29.3 ± 5.2^ab^	29.3 ± 3.9^a^
T6	56.1 ± 7.3^cd^	48.6 ± 6.5^de^	42.7 ± 9.6^de^	39.1 ± 5.4^bc^	27.2 ± 8.7^a^	37.5 ± 31.7^ab^
T7	62.7 ± 7.9^d^	50.9 ± 8.0^e^	48 ± 6.7^ef^	43.4 ± 12.6^cd^	35.1 ± 12.1^abc^	27.9 ± 15.2^a^
T8	62.6 ± 11.2^d^	48 ± 7.4^de^	51.2 ± 7.2^f^	47.2 ± 7.4^de^	40.3 ± 9.0^cd^	37.5 ± 14.0^ab^
T9	57.5 ± 10.1^cd^	51.6 ± 6.8^e^	48.3 ± 4.4^ef^	51.3 ± 4.9^e^	46.4 ± 11.4^d^	40.3 ± 10.6^ab^
T10	59.3 ± 11.0^cd^	48.2 ± 8.5^de^	51.1 ± 6.0^f^	49.9 ± 4.5^de^	46 ± 8.8^d^	49.7 ± 7.9^b^
T11	51.6 ± 12.8^bc^	28.3 ± 4.0^a^	34.6 ± 7.7^abc^	34.2 ± 5.5^ab^	37.6 ± 5.1^bc^	36.8 ± 5.7^ab^
STR						
T1	72.7 ± 6.9^bcd^	65.8 ± 8.3^c^	61.4 ± 5.0^c^	57.8 ± 16.5^a^	64.5 ± 10.7^cd^	57.3 ± 10.0^ab^
T2	66.4 ± 6.9^ab^	59.7 ± 7.4^b^	59.1 ± 5.9^bc^	56.8 ± 4.9^a^	51.7 ± 5.0^ab^	56.4 ± 8.1^ab^
T3	63.8 ± 7.0^a^	58.4 ± 7.1^b^	57.6 ± 6.1^abc^	55.3 ± 8.4^a^	51.2 ± 7.0^ab^	47.4 ± 12.5^a^
T4	62.8 ± 6.2^a^	57.4 ± 6.2^b^	54 ± 6.2^ab^	50.1 ± 5.7^a^	49.3 ± 6.4^a^	49.4 ± 9.1^a^
T5	64.2 ± 6.0^a^	56.4 ± 7.8^ab^	52.5 ± 5.7^a^	49.6 ± 6.2^a^	48.7 ± 5.9^a^	49.4 ± 6.1^a^
T6	74.3 ± 4.6^cd^	71.7 ± 4.3^d^	68.1 ± 8.3^d^	66.6 ± 4.7^b^	53.3 ± 22.1^ab^	58.5 ± 34.4^ab^
T7	78.5 ± 4.9^d^	75.3 ± 5.4^d^	70 ± 8.5^de^	69.2 ± 13.8^bc^	65.2 ± 15.8^cde^	51 ± 16.4^a^
T8	78.4 ± 7.2^d^	74.5 ± 5.4^d^	75.6 ± 6.1^ef^	73.7 ± 3.8^bcd^	70.4 ± 7.4^de^	69.3 ± 8.6^bc^
T9	75.3 ± 6.5^d^	76.9 ± 5.0^d^	74.5 ± 3.5^ef^	76.1 ± 3.7^cd^	75.2 ± 8.9^e^	69.7 ± 12.8^bc^
T10	76.4 ± 6.2^d^	73.9 ± 8.7^d^	77 ± 3.4^f^	77.4 ± 4.5^d^	73.5 ± 7.5^de^	76.2 ± 7.2^c^
T11	67.9 ± 10.0^abc^	50.4 ± 2.7^a^	56.1 ± 9.4^abc^	57.1 ± 5.0^a^	59.9 ± 4.4^bc^	55.3 ± 5.0^ab^
WOB						
T1	70.7 ± 8.4^abc^	64.9 ± 8.2^bc^	65.6 ± 8.9^bcd^	56.5 ± 10.4^a^	56.2 ± 14.1^ab^	50.7 ± 11.3^a^
T2	69.2 ± 7.2^ab^	67 ± 6.3^c^	65.8 ± 6.6^bcd^	65.5 ± 5.2^bc^	64.1 ± 7.5^b^	59.8 ± 7.3^ab^
T3	66.1 ± 4.9^a^	63.4 ± 7.2^bc^	66.4 ± 6.1^bcd^	63.4 ± 4.0^abc^	58.8 ± 4.6^ab^	62.7 ± 6.4^ab^
T4	66 ± 4.5^a^	60.1 ± 5.7^ab^	62.1 ± 5.6^abc^	60.3 ± 6.9^ab^	62.4 ± 5.8^b^	60.6 ± 4.9^ab^
T5	64.6 ± 6.8^a^	58.4 ± 4.1^ab^	58 ± 4.8^a^	59.2 ± 4.5^ab^	59.8 ± 4.9^ab^	59.2 ± 3.2^ab^
T6	75.3 ± 5.6^bcd^	67.6 ± 6.0^c^	62.1 ± 8.3^abc^	58.6 ± 5.8^ab^	56.3 ± 18.0^ab^	50.6 ± 33.8^a^
T7	79.6 ± 5.6^d^	67.3 ± 6.8^c^	68.4 ± 2.9^d^	61.5 ± 8.8^abc^	52 ± 10.5^a^	52.3 ± 14.0^ab^
T8	79.2 ± 9.5^d^	64.3 ± 6.7^bc^	67.5 ± 4.9^cd^	63.7 ± 7.7^bc^	56.7 ± 8.2^ab^	52.5 ± 15.6^ab^
T9	75.9 ± 7.8^bcd^	66.9 ± 6.3^c^	64.7 ± 3.6^bcd^	67.4 ± 6.6^c^	60.7 ± 10.5^ab^	57.2 ± 6.3^ab^
T10	77.1 ± 9.0^cd^	64.9 ± 5.4^bc^	66.3 ± 5.3^bcd^	64.5 ± 3.6^bc^	62.1 ± 7.6^ab^	65.1 ± 6.6^b^
T11	75.3 ± 8.3^bcd^	56.1 ± 6.2^a^	60.9 ± 4.6^ab^	59.5 ± 5.1^ab^	62.7 ± 7.0^b^	66.3 ± 7.9^b^

Different letter notations in the same column indicate a significant difference (p < 0.05). LIN=Linearity, STR=Straightness, WOB=Wobble

Table-4 lists the ALH, BCF, and H of spermatozoa in liquid semen during cold storage. The ALH value of spermatozoa in the diluent CEP-3(m) + ASE 1 was higher than CEP-3(m) + ASE 2, indicating a wide deviation of the head movement of the spermatozoa, potentially leading to a star-shaped pattern. The H value decreased during cold storage, and the value on days 1–5 in diluent CEP-3(m) + ASE 1 at level 30% was higher than spermatozoa in diluent CEP-3(m) + EY 10%. Meanwhile, the BCF value in the CEP-3(m) + ASE 2 diluent was the same as the positive control diluent and higher than the diluent with ASE 1.

**Table-4:** Amplitude lateral head, BCF, and H of spermatozoa liquid semen with various diluent formulas.

ALH	Days of cold storage					

	0	1	2	3	4	5
T1	2.8 ± 0.4^bc^	2.6 ± 0.4^abc^	2.1 ± 0.7^ab^	1.9 ± 0.7^b^	0.5 ± 0.3^a^	0.9 ± 1.0^a^
T2	3 ± 0.4^cd^	2.8 ± 0.4^c^	2.5 ± 0.3^cde^	2.4 ± 0.3^bcd^	2.1 ± 0.7^bc^	1.7 ± 0.9^b^
T3	3.3 ± 0.3^d^	3 ± 0.2^d^	2.8 ± 0.4^def^	2.6 ± 0.1^cde^	2.4 ± 0.5^bc^	1.8 ± 1.0^b^
T4	3.3 ± 0.3^d^	3.3 ± 0.4^ef^	3.1 ± 0.4^f^g	2.8 ± 0.4^de^	2.7 ± 0.6^c^	2.4 ± 0.4^bc^
T5	3.2 ± 0.4^d^	3.4 ± 0.3^f^	3.3 ± 0.3g	3 ± 0.4^e^	2.7 ± 0.4^c^	2.6 ± 0.5^c^
T6	2.5 ± 0.3^ab^	2.4 ± 0.3^a^	2 ± 0.7^a^	1.3 ± 0.9^a^	0.7 ± 0.7^a^	0.2 ± 0.6^a^
T7	2.3 ± 0.3^a^	2.5 ± 0.2^ab^	2.1 ± 0.3^ab^	2.1 ± 0.8^bc^	1.8 ± 1.0^b^	0.4 ± 0.6^a^
T8	2.3 ± 0.5^a^	2.6 ± 0.2^abc^	2.3 ± 0.2^abc^	2.2 ± 0.2^bc^	1.8 ± 0.6^b^	1.7 ± 0.7^b^
T9	2.4 ± 0.4^a^	2.6 ± 0.2^abc^	2.4 ± 0.2^bcde^	2.3 ± 0.4^bc^	2.1 ± 0.9^bc^	2.2 ± 0.7^bc^
T10	2.3 ± 0.4^a^	2.7 ± 0.2^bc^	2.4 ± 0.3^bcd^	2.3 ± 0.2^bc^	2.1 ± 0.5^bc^	2 ± 0.5^bc^
T11	2.4 ± 0.4^a^	3.2 ± 0.2^de^	2.8 ± 0.2^ef^	2.8 ± 0.2^de^	2.7 ± 0.4^c^	2.2 ± 0.3^bc^
BCF						
T1	9.6 ± 1.1^b^	10.9 ± 1.5^bcde^	8.3 ± 2.6^ab^	8.9 ± 4.4^b^	3.3 ± 2.0^a^	2.7 ± 3.2^a^
T2	8.3 ± 1.5^ab^	10.5 ± 1.2^bcd^	9.5 ± 1.5^abc^	9.5 ± 1.2^bc^	7.8 ± 2.3^bc^	6.8 ± 3.6^b^
T3	8.4 ± 1.5^ab^	9.9 ± 1.4^ab^	9.6 ± 2.1^abc^	9.6 ± 1.2^bcd^	8.3 ± 1.0^bcd^	7.2 ± 3.9^b^
T4	8.1 ± 1.4^ab^	9 ± 1.5^a^	9.2 ± 1.6^ab^	8.6 ± 1.0^b^	8.8 ± 1.6^bcd^	8.4 ± 1.3^bc^
T5	7.8 ± 1.7^a^	9.1 ± 1.1^a^	8 ± 1.3^a^	8.5 ± 1.2^b^	8.5 ± 0.7^bcd^	8.5 ± 1.3^bc^
T6	9.1 ± 1.5^ab^	10.9 ± 0.8^bcde^	9.2 ± 3.0^ab^	6.3 ± 4.3^a^	1.9 ± 2.4^a^	1.2 ± 3.0^a^
T7	9.2 ± 1.8^ab^	12 ± 1.2^e^	11.2 ± 1.5^cde^	9.1 ± 3.9^b^	7.4 ± 3.5^b^	2.5 ± 3.0^a^
T8	9.5 ± 2.0^ab^	11.7 ± 1.3^de^	11.5 ± 1.4^de^	11.5 ± 1.1^cde^	9 ± 3.7^bcd^	7.9 ± 3.9^b^
T9	8.7 ± 2.0^bc^	11.6 ± 1.6^de^	11.4 ± 1.1^de^	12.2 ± 0.8^e^	10.5 ± 4.0^cd^	8.9 ± 3.1^bc^
T10	8.8 ± 1.7^ab^	11.4 ± 1.6^cde^	12.2 ± 1.3^e^	11.9 ± 0.8^de^	11.1 ± 2.6^d^	11.5 ± 3.4^c^
T11	7.8 ± 1.6^a^	10 ± 1.0^abc^	9.8 ± 1.2^bcd^	10 ± 0.9^bcde^	9.8 ± 0.6^bcd^	9.5 ± 1.0^bc^
H						
T1	23.5 ± 6.6^cd^	9.7 ± 6.2^ab^	2.7 ± 1.8^ab^	1.5 ± 1.6^a^	0.1 ± 0.2^a^	1.1 ± 1.4^a^
T2	21.1 ± 3.9^bc^	9.8 ± 3.1^ab^	6.9 ± 3.6^abc^	2.9 ± 2.6^a^	1.7 ± 2.0^a^	1.3 ± 0.9^a^
T3	27 ± 9.0^cd^	17.2 ± 5.9^de^	11 ± 4.3^cde^	6.7 ± 2.6^b^	4.5 ± 3.1^a^	0.9 ± 0.7^a^
T4	26.8 ± 10.4^cd^	19.6 ± 5.5^de^	15.8 ± 6.8^ef^	11.6 ± 3.4^c^	9.7 ± 7.2^b^	5.7 ± 3.0^b^
T5	28.4 ± 4.7^d^	20.8 ± 5.7^e^	18.3 ± 8.7^f^	14.9 ± 5.6^d^	13.8 ± 8.1^c^	10.6 ± 8.0^c^
T6	15.7 ± 3.2^ab^	6.1 ± 3.9^a^	1.9 ± 2.2^a^	0.4 ± 0.6^a^	0.1 ± 0.2^a^	0.2 ± 0.5^a^
T7	15.3 ± 5.9^ab^	11.6 ± 5.1^bc^	3.3 ± 2.4^abc^	2.2 ± 2.0^a^	0.4 ± 0.5^a^	0.1 ± 0.1^a^
T8	14 ± 5.5^a^	15 ± 1.8^cd^	7.1 ± 2.9^bc^	1.7 ± 1.1^a^	0.5 ± 0.6^a^	0.6 ± 1.0^a^
T9	14.6 ± 5.0^a^	17.7 ± 5.6^de^	10.3 ± 5.9^cd^	6.3 ± 5.0^b^	2.4 ± 2.2^a^	1.1 ± 1.6^a^
T10	14.8 ± 4.8^a^	17.3 ± 4.8^de^	12.7 ± 7.1^de^	7.3 ± 3.9^b^	3.4 ± 3.2^a^	1.3 ± 1.6^a^
T11	12.8 ± 4.6^a^	11.7 ± 2.5^bc^	12 ± 2.4^cde^	10.9 ± 1.4^c^	9.5 ± 2.4^b^	5.3 ± 2.9^b^

Different letter notations in the same column indicate a significant difference (p < 0.05). ALH=Amplitude lateral head, BCF=Beat cross frequency, H=Hyperactivity

Based on Table-5, the diluents CEP-3(m) + EY 10% and CEP-3(m) + ASE 1 (25%–30%) can support the life of spermatozoa (viability) up to day 5 with a value exceeding 80% (81.8 ± 3.5%; 86.4 ± 2.6%). The viability of spermatozoa in the diluent with ASE 1 was better than in the diluent with ASE 2. The abnormality value of spermatozoa in various diluents (CEP-3[m] and ASE 1 and 2) during cold storage on days 0–5 was below 20%.

**Table-5:** Viability and abnormality of spermatozoa liquid semen with various diluent formulas.

Viability	Days of cold storage					

	0	1	2	3	4	5
T1	85.4 ± 5.2^b^	75 ± 11.6^ab^	66 ± 9.2^ab^	65.4 ± 20.5^abc^	49.6 ± 19.1^a^	33.8 ± 17.3^a^
T2	85.8 ± 6.1^b^	84.4 ± 10.4^b^	81 ± 10.6^cd^	70 ± 14.6^abc^	66 ± 11.6^a^	54.6 ± 9.1^bc^
T3	90 ± 4.1^b^	86 ± 5.5^b^	86.4 ± 4.2^cd^	84 ± 4.3^c^	76.8 ± 5.5^bc^	71 ± 10.0^cd^
T4	85.8 ± 4.0^b^	87.4 ± 7.4^b^	81.4 ± 7.8^cd^	83.2 ± 4.6^c^	81.6 ± 8.0^bc^	81.8 ± 3.5^d^
T5	85.4 ± 6.9^b^	86.2 ± 8.8^b^	90.4 ± 6.7^d^	73.6 ± 17.8^abc^	87.6 ± 4.0^c^	86.4 ± 2.6^d^
T6	72.2 ± 19.0^a^	68 ± 12.5^a^	63.8 ± 13.9^a^	57.2 ± 13.0^ab^	47.6 ± 19.1^a^	37.6 ± 16.5^a^
T7	80 ± 8.5^ab^	77 ± 16.3^ab^	72.8 ± 14.3^abc^	53.4 ± 27.1^a^	50.4 ± 26.6^a^	46.4 ± 24.4^ab^
T8	83.2 ± 11.1^ab^	79.8 ± 11.3^ab^	78.2 ± 12.0^bcd^	74.2 ± 6.8^abc^	71.2 ± 10.1^bc^	55.2 ± 20.8^bc^
T9	85.2 ± 6.2^b^	84.8 ± 6.1^b^	80.2 ± 6.5^cd^	76.6 ± 11.4^bc^	64.6 ± 15.2^ab^	57.8 ± 19.3^bc^
T10	83.8 ± 7.8^ab^	88.2 ± 6.1^b^	86.4 ± 5.6^cd^	78.8 ± 9.7^bc^	77 ± 7.7^bc^	71.6 ± 13.8^cd^
T11	90.8 ± 6.4^b^	86.8 ± 13.3^b^	88.8 ± 5.8^d^	87 ± 6.2^c^	87.8 ± 3.0^c^	83.8 ± 1.7^d^
Abnormality						
T1	1.6 ± 0.9	0.8 ± 0.8	0.8 ± 1.3	0.8 ± 0.8	1.4 ± 1.3	0.6 ± 0.9^ab^
T2	1.2 ± 0.8	1 ± 1.7	0.8 ± 0.8	1.4 ± 1.1	1.2 ± 2.2	1.4 ± 1.1^ab^
T3	1.2 ± 0.8	1.4 ± 1.5	0.2 ± 0.4	0.8 ± 0.8	1 ± 1.2	1.2 ± 1.1^ab^
T4	2 ± 0.7	1.2 ± 0.8	1.8 ± 2.9	1.6 ± 1.3	1.6 ± 1.5	1.4 ± 1.7^ab^
T5	1.2 ± 1.8	1.8 ± 0.8	0.8 ± 0.8	1.8 ± 1.6	1.6 ± 1.9	1 ± 1.0^ab^
T6	1.6 ± 1.1	1.4 ± 1.1	0.8 ± 1.1	0.8 ± 0.8	0.6 ± 0.5	0.2 ± 0.4^a^
T7	2 ± 1.4	1.8 ± 1.3	1 ± 1.4	1.2 ± 0.8	2.2 ± 2.2	0.4 ± 0.5^ab^
T8	1 ± 1	1.8 ± 2.4	1.2 ± 1.8	1 ± 1.2	1 ± 1.2	1.6 ± 0.9^ab^
T9	1.2 ± 0.8	1.6 ± 1.7	1 ± 1.4	0.8 ± 1.1	1.4 ± 1.1	2.2 ± 1.9^b^
T10	1.8 ± 2.7	1.4 ± 0.5	0.6 ± 0.9	0.6 ± 0.9	1.8 ± 1.3	2 ± 1.6^ab^
T11	1.8 ± 2.2	1.5 ± 1	0.5 ± 1	1.3 ± 1.2	2.5 ± 1	1.8 ± 1.3^ab^

Different letter notations in the same column indicate a significant difference (p < 0.05)

**Figure-1:**
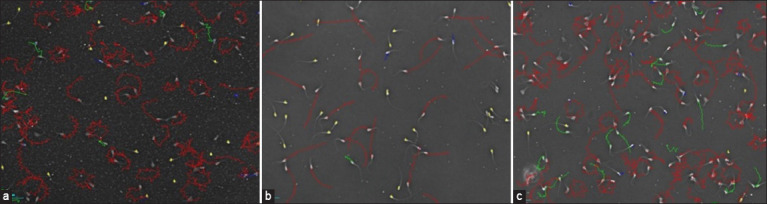
Spermatozoa trajectory pattern during cold storage in diluent (a) (CEP-3[m] + aqueous soybean extract [ASE] 1 at 30%), (b) (CEP-3[m] + ASE 2 at 30%), and (c) (CEP-3[m] + Egg Yolk at 10%).

Based on Figures-1a and c, it is evident that the trajectory pattern of spermatozoa in CEP-3(m) diluent and ASE 1 was almost identical to that of CEP-3(m) diluent and 10% EY. Most track patterns of spermatozoa were circular and non-progressive. However, the immotile spermatozoa in the diluent exhibited a lower percentage than the spermatozoa in the CEP-3(m) diluent and ASE 2. Meanwhile, the spermatozoa in the diluent CEP-3(m) and ASE 2 mostly showed a more progressive spermatozoa trajectory pattern, but some spermatozoa with a circular trajectory were also observed. The percentage of immotile spermatozoa in this diluent was higher than that of the CEP-3(m) diluent and ASE 1. Immotile spermatozoa were detected in yellow color, but this did not necessarily indicate dead spermatozoa.

## Discussion

Motility is an important parameter indicating the potential fertilization of spermatozoa, membrane integrity, and viability. Spermatozoa M analysis using CASA resulted in a more accurate assessment [38]. Based on the result, spermatozoa in CEP-3(m) diluent and ASE 1 showed PM >40% until day 5, while in CEP-3(m) diluent and ASE 2, it only lasted until the 3^rd^ day. This suggests that ASE 1 provides better protection against cold shock. There is a gradual decrease in M during cold storage due to the depletion of energy sources for spermatozoa metabolism and the effect of lipid peroxidation (reactive oxygen species), which damages the spermatozoa membranes [39]. Progressive motility indicates the percentage of motile spermatozoa with a VAP of more than 25 m/s. Meanwhile, spermatozoa M is the percentage of mobile spermatozoa [40]. Motility plays an essential role in the passage of spermatozoa to the oocyte and their penetration into the zona pellucida. It determines the vitality, metabolism status of spermatozoa, and the status of its cell membranes. Motile spermatozoa indicate intact cell membranes, while immotile ones indicate damaged membranes [39].

A decrease in M also contributes to a decrease in the speed of spermatozoa movement (velocity) during cold storage. There were similarities in the VCL values in the CEP-3(m) diluent and ASE 1 and the positive control. The VCL value shows the vigor or strength of the spermatozoa movement and their speed in a curved trajectory pattern but does not indicate progressivity. Meanwhile, spermatozoa in CEP-3(m) diluent and ASE 2 had a higher VSL value than ASE 1 and the positive control. The VSL value indicated the speed of movement of spermatozoa in a straight line. Several factors affect the velocity and M of spermatozoa, including the energy source of spermatozoa, viscosity, osmolarity, and pH. Based on the results of visual observations using CASA, it is evident that the movement of spermatozoa in the CEP-3(m) diluent and ASE 1 at the 30% level showed that many spermatozoa tended to rotate (Figure-1a). This is also supported by lower LIN and STR values and higher ALH values than spermatozoa in CEP-3(m) diluent and ASE 2. The resulting movement pattern is star-shaped, indicating that spermatozoa have H.

Hyperactivity is a condition in which spermatozoa move very quickly, but are not linear and progressive and tend to move around. Some of the causes of hyperactive spermatozoa include a surplus of energy sources and an indication of capacitation [41]. In this case, it is suspected that the cause was an increase in the viscosity of the CEP-3(m) diluent and ASE 1. The thicker solution makes it difficult for the spermatozoa to move progressively, causing them to rotate. The CEP-3(m) diluent formula and ASE 1 visually observed through CASA showed a lot of debris or vacuole around the spermatozoa, which further hampered their progressivity. Soybean extraction method 1 is a hot grinding method, which is a modified extraction method [28, 32].

The formula diluent CEP-3(m) and ASE- 2 at a 30% level showed that the solution was not too viscous, so the spermatozoa had no difficulty in moving, and very minimal debris was observed. During cold storage, spermatozoa in the CEP-3(m) diluent and ASE 2 showed a more regular and progressive movement/trajectory pattern (Figure-1b), including higher VSL, LIN, STR, BCF, and ALH, H values lower than in ASE 1. The disadvantage of ASE 2 is vigor, as the M of spermatozoa was not as strong as in ASE 1. The CEP-3 (m) diluent formula with a higher level of ASE 2 has the potential to produce better performance, thereby requiring further studies.

The protective effect of extracted soybean can also be determined by the percentage of spermatozoa viability during cold storage. Spermatozoa viability above 80% can be maintained in CEP-3(m) diluent and ASE 1 at the 25%–30% level until the 5^th^ day. However, the CEP-3(m) diluent and ASE 2 can only last until the 2^nd^ day. This was also supported by the decreasing value of spermatozoa M during cold storage at various levels of ASE 2. There was a slight increase in the value of spermatozoa abnormalities on the 5^th^ day of storage, but overall, it was still within normal limits. Abnormalities during cooling can occur due to various physical and chemical treatments during storage. Another possibility, it was caused by low livestock genetic factors [36].

Every extraction method produces soybean extract with different compositions and characteristics. Soy lecithin can be extracted mechanically or chemically. Various soybean extraction methods have been developed previously, including water extraction, Pre-blanch Methode, and Hot Grinding extraction, which yield soya milk (soy milk). Soybean preparation before grinding includes the process of pre-soaking or without soaking, with or without the addition of NaHCO_3_ (Pre-blanch Methode). Grinding can be done with cold or hot water (Hot Grinding Method). Mechanical extraction can be done through milling or extraction [14].

## Conclusion

Based on the results, soybean extracts 1 and 2 have the potential to serve as extracellular cryoprotectants to substitute EYs in modified CEP-3 basic diluents. Soybean extract 1 was found to support the life of spermatozoa up to the 5^th^ day, although there are limiting factors, such as the viscosity and hyperactivity of spermatozoa. Soybean extract 2 can sustain spermatozoa until the 3^rd^ day of cold storage and produces progressive, non-rotating movement patterns. Further, research is necessary to explore the potential of higher levels of soybean extract 2.

## Authors’ Contributions

DR: Contributed the concept and design of the study, collected data, analyzed data, and reviewed the final version of the manuscript. KK, APAY: Contributed the concept, designed the study, and reviewed the manuscript. GC, SR and TS: Designed the study, supervision of the study, and reviewed the manuscript. All authors have read, reviewed, and approved the final manuscript.
